# Rare variant burden analysis from exomes of three consanguineous families reveals *LILRB1* and *PRSS3* as potential key proteins in inflammatory bowel disease pathogenesis

**DOI:** 10.3389/fmed.2023.1164305

**Published:** 2023-05-05

**Authors:** Rana Mohammed Jan, Huda Husain Al-Numan, Nada Hassan Al-Twaty, Nuha Alrayes, Hadeel A. Alsufyani, Meshari A. Alaifan, Bakr H. Alhussaini, Noor Ahmad Shaik, Zuhier Awan, Yousef Qari, Omar I. Saadah, Babajan Banaganapalli, Mahmoud Hisham Mosli, Ramu Elango

**Affiliations:** ^1^Department of Biological Sciences, Faculty of Science, King Abdulaziz University, Jeddah, Saudi Arabia; ^2^Princess Al-Jawhara Al-Brahim Center of Excellence in Research of Hereditary Disorders, King Abdulaziz University, Jeddah, Saudi Arabia; ^3^Department of Medical Laboratory Sciences, Faculty of Applied Medical Sciences, King Abdulaziz University, Jeddah, Saudi Arabia; ^4^Department of Medical Physiology, Faculty of Medicine, King Abdulaziz University Hospital, Jeddah, Saudi Arabia; ^5^Department of Pediatrics, Faculty of Medicine, King Abdulaziz University, Jeddah, Saudi Arabia; ^6^Department of Genetic Medicine, Faculty of Medicine, King Abdulaziz University, Jeddah, Saudi Arabia; ^7^Department of Clinical Biochemistry, Faculty of Medicine, King Abdulaziz University, Jeddah, Saudi Arabia; ^8^Department of Internal Medicine, King Abdulaziz University, Jeddah, Saudi Arabia; ^9^Inflammatory Bowel Disease Research Group, King Abdulaziz University, Jeddah, Saudi Arabia

**Keywords:** inflammatory bowel disease, missense mutation, Crohn’s disease, gastrointestinal tract, protein modeling

## Abstract

**Background:**

Inflammatory bowel disease (IBD) is a chronic autoimmune disorder characterized by severe inflammation and mucosal destruction of the intestine. The specific, complex molecular processes underlying IBD pathogenesis are not well understood. Therefore, this study is aimed at identifying and uncovering the role of key genetic factors in IBD.

**Method:**

The whole exome sequences (WESs) of three consanguineous Saudi families having many siblings with IBD were analyzed to discover the causal genetic defect. Then, we used a combination of artificial intelligence approaches, such as functional enrichment analysis using immune pathways and a set of computational functional validation tools for gene expression, immune cell expression analyses, phenotype aggregation, and the system biology of innate immunity, to highlight potential IBD genes that play an important role in its pathobiology.

**Results:**

Our findings have shown a causal group of extremely rare variants in the *LILRB1* (Q53L, Y99N, W351G, D365A, and Q376H) and *PRSS3* (F4L and V25I) genes in IBD-affected siblings. Findings from amino acids in conserved domains, tertiary-level structural deviations, and stability analysis have confirmed that these variants have a negative impact on structural features in the corresponding proteins. Intensive computational structural analysis shows that both genes have very high expression in the gastrointestinal tract and immune organs and are involved in a variety of innate immune system pathways. Since the innate immune system detects microbial infections, any defect in this system could lead to immune functional impairment contributing to IBD.

**Conclusion:**

The present study proposes a novel strategy for unraveling the complex genetic architecture of IBD by integrating WES data of familial cases, with computational analysis.

## 1. Introduction

Inflammatory bowel disease (IBD) is a chronic immune disorder characterized by severe inflammation and mucosal destruction in the colon and small intestine (1, 2). Crohn’s disease (CD) and ulcerative colitis (UC) are the two major forms of IBD, which share identical pathological and clinical symptoms (2, 3). However, each condition shows a variable clinical presentation, response to treatment, and genetic risk factors (3). Recent decades have seen a sharp increase in the prevalence of IBD, which could be attributed to industrialization and lifestyle changes. The high prevalence of consanguinity in the Arab population results in the perpetuation of numerous harmful genetic variants in society. This aggregation of damaging variants in key genes may cause rare monogenic diseases and increase the genetic contribution to complex diseases such as IBD. Although the primary cause of IBD is unknown, interactions between environmental and immunoregulatory variables have been identified as a probable cause in genetically predisposed individuals (4, 5).

There is a clear evidence that genetic factors play an important role, with relatives of UC and CD patients having 8- to 10-fold increased risk of developing IBD (6). The strongest evidence for a genetic predisposition to IBD came from twin studies. While genetic defects in the IL-10 signaling pathway have been identified as an underlying molecular cause for very-early-onset IBD (VEO-IBD), no single causal genetic factor has been identified for late-onset IBD. This is because, late-onset IBD has a polygenic etiology, and environmental factors determine the susceptibility and age of onset of the disease (7). However, genome-wide association studies (GWAS) have uncovered more than 200 common risk loci in IBD pathogenesis (8–12). Some of these risk alleles are missense variants that have been mapped to genes such as Interleukin 23 Receptor (*IL23R*), Nucleotide Binding Oligomerization Domain containing 2 (*NOD2*), and Autophagy-related 16 like 1 (*ATG16L1*) (13). Majority of these risk markers are intronic variants (14).

Thus, to better understand the pathogenesis of complex diseases, application of next-generation sequencing technologies is having a greater impact, especially in consanguineous societies (15–17). They will provide an excellent opportunity to identify rare variants with intermediate to high effect ranges more efficiently. These rare variants are believed to have high odds ratios (ORs) and high penetrance and are suitable for functional experimental validation. In genetics, OR is often used to quantify the risk of developing a particular disease in individuals who carry a specific genetic variant or mutation. In a recent study, one rare coding variant in the *BTNL2* gene within the Major histocompatibility complex (MHC) region was associated with higher IBD risk (OR-2.3), giving an insight into T cell activation mechanisms and IBD sub-phenotype developments (18). It provides strong support for our planned approach to identify potential causal variants and genes for IBD through familial studies. Since published information on the genetics of Arab IBD familial patients is limited, the goal of this study is to find out the causal genetic variants involved in IBD pathogenesis.

## 2. Materials and methods

### 2.1. Recruitment of families with IBD

The Biomedical Ethics Research Committee of King Abdulaziz University Hospital in Jeddah (KAUH) approved the proposed research project. At the Internal Medicine specialty gastroenterology clinics at King Abdulaziz University Hospital, Jeddah (KAUH), three unrelated Saudi consanguineous families with many affected siblings, who fulfilled the inclusion criteria of the study, reporting abdominal pain along with weight loss and persistent diarrhea, were recruited. An informed consent to join the research as participants was signed by all family members before we collected clinical data and blood samples. Family A has two siblings with IBD, and families B and C each have three siblings with IBD. All these patients were examined by a consultant gastroenterologists, and the diagnosis was arrived at as per the standard diagnostic criteria set out by the European Crohn’s and Colitis Organization (ECCO) 2019 (19). After collecting the full family history, a three-generation pedigree for each family was constructed. Hospital electronic health records were accessed to collect clinical history on all affected siblings. For genetic analysis, approximately 3–4 mL of peripheral blood was collected in EDTA tubes from all participants and stored at −80°C until used.

### 2.2. DNA purification

Genomic DNA was purified according to the manufacturer’s instructions using the QIAamp DNA Blood Kit (Qiagen, United States). A Nanodrop (ND-1000 UV–VIS) spectrophotometer was used to measure DNA concentration and purity. The DNA integrity for high molecular weight DNA was evaluated using 1% agarose gel electrophoresis, and the gel image was captured in a UV transilluminator attached camera. All the samples were stored at −20°C until they were used for genetic analysis.

### 2.3. Whole exome sequence analysis

Whole exome sequencing was performed using the Illumina HiSeq2000 next-generation sequencer (Illumina Inc., San Diego, CA, United States). The whole exome-enriched library was constructed using genomic DNA at an average concentration of 60 ng/μL, including DNA tagmentation (fragmentation and adapter ligation at both ends), target capturing, and amplification using the ligated adapters. The Agilent SureSelect exome capture kit V7.0 (Agilent Technologies, United States) was used to shear all exonic sections of protein-coding genes that were registered in the CCDS and RefSeq databases, resulting in ideal size-range fragments. Ultra-long 120-mer biotinylated cRNA library baits were used to hybridize the fragmented DNA. Capillary electrophoresis was used to determine the concentration and size of the library. During enrichment, various adapters were incorporated, allowing the samples to be amplified for subsequent sequencing. For variant calling and annotation, the sequencing reads (in the FASTQ format) were matched to the human genome reference sequence build 38 (GRCH38.p12) using BLAST (version 0.6.4d). Variants were filtered based on the following criteria: depth (30), maximum quality read (60), alternative to total depth ratio (>80% for homozygous variants and 40–70% for heterozygous variants), minor allele frequency (<0.01) based on the 1,000 genomes, gnomAD database, and location (coding regions or regulatory sites). The rare variants were further filtered based on the segregation pattern of the variants under different genetic inheritance models such as autosomal recessive (AR), compound heterozygous (CH), and *de novo* to identify the disease-causing variants.

#### 2.3.1. Identifying the rare variant burden genes

Since IBD is a complex disease with polygenic involvement, we tried to identify the genes with a rare variant burden. From the exome sequencing data of individual families, we attempted to identify genes harboring rare variants to see which genes are potentially involved in the disease causation.

### 2.4. Functional enrichment analysis using immune pathways

The rare variant harboring genes shared between the three families were initially identified by the Venny 2.1.0 web tool.^1^ The ClueGo, a Cytoscape plug-in was then used to perform functional enrichment analysis on these rare variant genes. For pathway enrichment of query genes, the GO annotations was chosen in the ClueGo settings (6). In this enrichment test, default stringent statistical options, such as Bonferroni multiple testing correction and enrichment/depletion (Two-sided hypergeometric test), were applied. The common pathways (enriched GO terms) among all three families were identified by the VENNY tool. The pathways corresponding to the mapped genes with rare variants that were shared by all three families were then further filtered to exclude contributing genes that were not included in the initial query list of shared rare variant genes.

### 2.5. Computational functional validation of selected potential IBD genes

The shared genes with rare variants from the pathway analysis were further filtered to validate their potential contribution to disease development. To this end, several databases were used to explore their gene expression levels in different organs and to prioritize the potential therapeutic drug targets and disease phenotype annotations.

#### 2.5.1. Gene expression analysis and exome validation

We examined the changes in the expression status of our query genes in IBD tissues by downloading 24 IBD-related transcript expression datasets hosted in Expression Atlas.^2^ This database is maintained by the European Bioinformatics Institute and provides information on gene expression patterns from RNA-seq, microarray studies, and protein expression from proteomics studies. The keywords searched in the database were IBD and inflammation. Different experimental samples were used, such as colonic, mucosal biopsies and peripheral blood monocytes, for different diseases such as UC, IBD, CD, irritable bowel syndrome, colorectal cancer, and colon adenomas. From the resultant datasets, we identified differentially expressed genes (DEGs) using a logFC cutoff fold change of >1 at *p* < 0.05. Furthermore, the EBI gene expression atlas (GXA) interface in Ensembl was used to search for transcript expression data of the query genes in different organs and tissues. The input is the gene name, and the output is the baseline expression in transcripts per million (TPM). Only the expression data of query genes (>0.5 TPM cutoff value) in the gastrointestinal tract, immunological organs, and blood were chosen from the output.

#### 2.5.2. Immune cell expression analysis

The Database of Immune Cell Expression (DICE)^3^, expression quantitative trait loci (eQTLs), and epigenomics were used to reveal the effect of IBD risk-associated genetic polymorphisms on specific immune cell types which might influence disease pathogenesis. This database delivers comprehensive information on immune cell expression generated by 15 immune cell types (subsets of T cells, B cells, monocytes, and NK cells). The input is the query gene ID, and the output is the expression level of genes in transcripts per million (TPM) on the *x*-axis, and cell types are sorted based on the *y*-axis of box plot graphs.

#### 2.5.3. Open target phenotype identification

The query hub genes were further analyzed using the Open Targets Platform.^4^ This website accesses several databases to help in clarifying the causal relationships between enzymatic reactions, physical binary interactions, or functional relationships between disease phenotypes and therapeutic targets (6). The input is the query gene list, and the output is the evidence score for a given target-disease pair. The significant value was set at a 0.5 cutoff score to detect the druggable molecular targets.

#### 2.5.4. System biology of innate immunity

The innate immunity interactions for the query genes were further explored by using the InnateDB website.^5^ This publicly available database with an integrated platform facilitates the systems-level analysis of innate immunity networks, pathways, and genes (20). The input is the gene name, and the output is the interactions and signaling responses involved in innate immunity processes.

### 2.6. Interaction gene networks and function prediction

The GeneMANIA plugin from Cytoscape was used to identify gene interaction networks from query genes and predict the gene’s putative function and annotation. The plugin uses a large database of functional interaction networks from *Homo sapiens*, and each related gene is traceable to the source network used to make the prediction. The input is the query gene list and the organism type. The output is a network of interconnected genes (21, 22).

### 2.7. Amino acid conserved domains

The functional relevance of rare genetic variants on candidate proteins was predicted by comparing the nucleotide and amino acid sequences to the functional domains of the concerned protein as listed in the Conserved Domain Database (CDD). CDD program uses RPS-BLAST, which efficiently scans the query protein for pre-computed position-specific score matrices (PSSMs), to estimate the sequence conservation characteristics of the functional domains of the candidate protein. Protein domains annotated with query input sequence and imagining options are shown in the output file.

### 2.8. Protein structure analysis

#### 2.8.1. Protein modeling and stability analysis

The Artificial Intelligence (AI) program developed by Alphabet/Google DeepMind, AlphaFold, generated protein structure at the molecular level^6^, which was extensively used to study the structural effect of the variants on the candidate proteins. The input is the protein, gene name, or UniProt accession, and organism name. The output is a predicted 3D protein model from its amino acid sequence with high accuracy (including side chains), a per residue confidence metric (PLDDT) that is used to color the residues of the prediction, and a predicted aligned error that is necessary to assess confidence in the domain packing and large-scale topology of the protein. The I-TASSER web tool was also used along with AlphaFold for the generation of protein structures that were not available in AlphaFold. I-TASSER predicts the 3D structure and biological activity of protein molecules based on their amino acid sequences using high-quality model predictions. The input is the amino acid sequence, and the output is several full-length atomic models along with their estimated accuracy (including a confidence score for all models, predicted TM-score, and RMSD for the first model), GIF images of the predicted models, and predicted secondary structure and solvent accessibility. To generate mutant protein models, SWISS-MODEL, a fully automated protein structure homology-modeling tool, was used. The input is the mutated amino acid sequence along with the wild-type template file in PDB format. Outputs include the 3D structure of models, their target–template sequence alignment, and model coordinates. The protein model PDB file is viewed by a molecular visualization system, PyMOL 2.5.^7^ PyMOL represents the protein in a three-dimensional (3D) model and is capable of editing molecules.

#### 2.8.2. Structural deviation and stability findings

The structural deviation between optimized native and variant protein models was determined using YASARA, a molecular graphics, modeling, and simulation tool. Two protein atomic coordinates were superimposed on top of each other, and the corresponding RMSD values were calculated to quantify structural similarity at both the global and local residue levels. The cut-off RMSD values for variant-induced structure deviations at the polypeptide chain and residue levels were > 0.2 and > 2, respectively. The effect of a candidate variant on protein structure stability was determined using the MAESTRO webserver. MAESTRO provides a confidence estimation C_pred_ for its total predicted change in stability (kcal/mol) ΔΔG predictions. ΔΔGpred <0.0 indicates a stabilizing mutation and C_pred_ is given as a value between 0.0 (not reliable) and 1.0 (highly reliable).

## 3. Results

### 3.1. Clinical and family history

**Figure 1:**
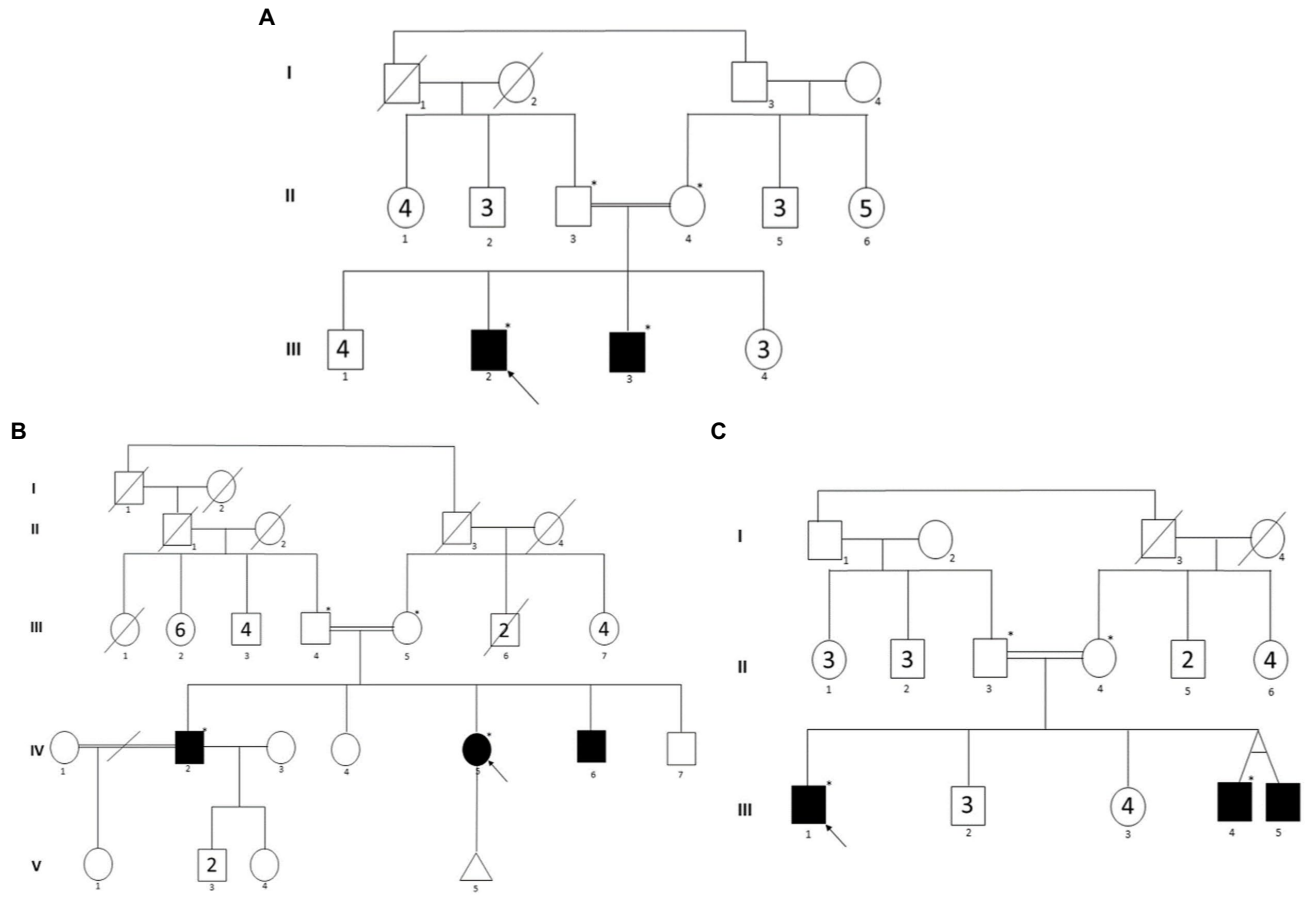
Three-generation pedigrees of families with IBD. **(A–C)** Represent the pedigrees of families A, B, and C, respectively. The black color circles or boxes represent patients with IBD. The arrow represents the proband in each family. The star represents individuals selected for WES.

**Figure 2:**
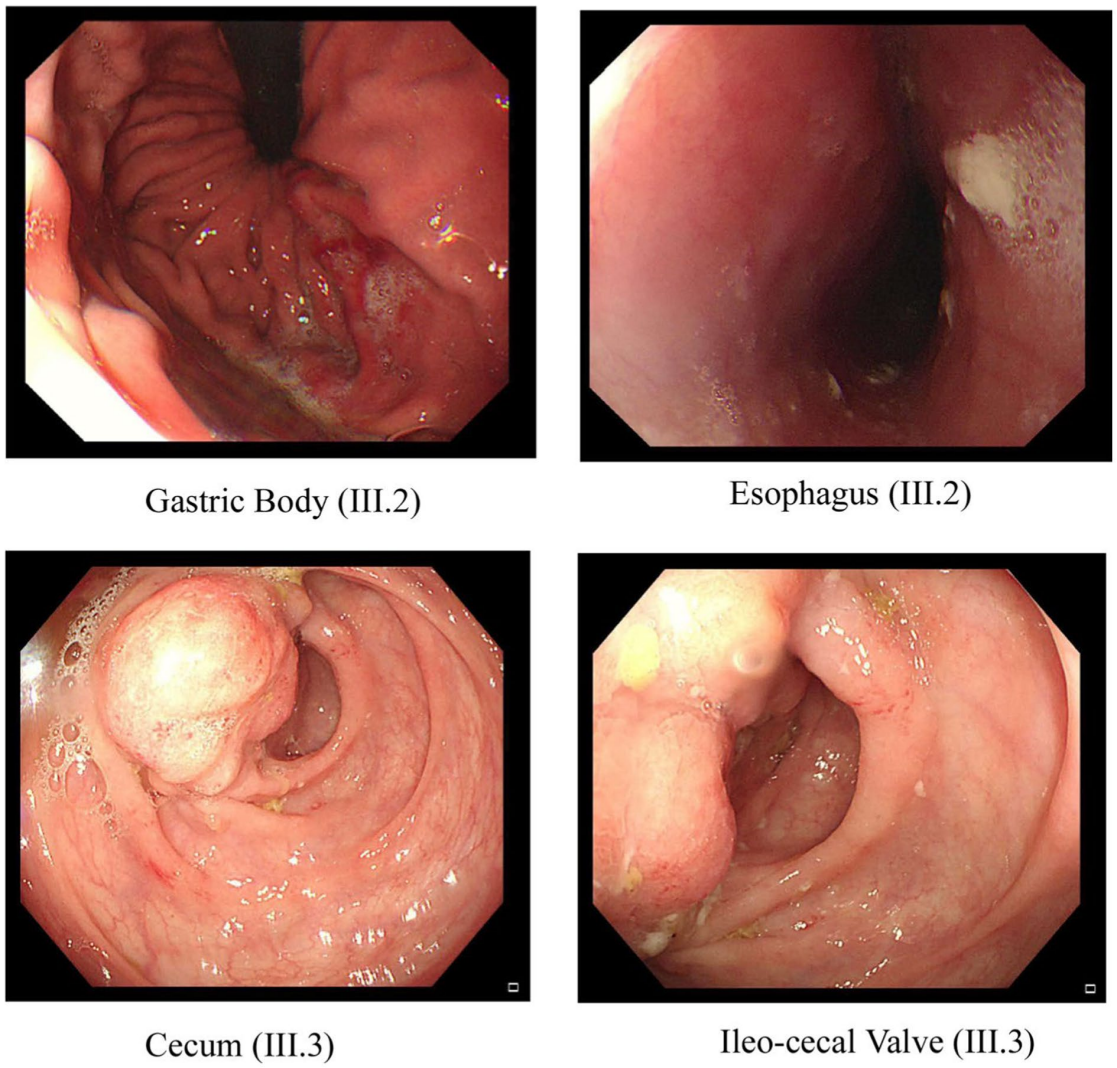
Endoscopic images of the GI tract of the proband III.2 and affected sibling III.3 in family A. Two pictures of the top row from proband III.2 show inflammation and ulceration lesions in the gastric body and eroded, punctate white spotted mucosa in the esophagus with a hemorrhagic gastropathy. Bottom row images from III.3 show inflammation and ulceration lesions in the cecum and ileocecal valve.

In family A (Figure 1A), the proband (III.2), aged 27 years, is the offspring of a consanguineous marriage between first cousins and has no family history of inflammatory bowel disease. He was first diagnosed with Crohn’s disease at the age of 22 years, suffering from several symptoms such as nausea, anorexia, and night sweats. His endoscopy test findings confirmed the diagnosis of Crohn’s disease with an eroded, punctate, white-spotted mucosa in the esophagus and a hemorrhagic gastropathy (Figure 2). He is currently being treated with Pentaza (mesalamine), which is a 5-aminosalicylic acid derivative, and Imuran (azathioprine AZA), an immunosuppressive medication. His younger brother (III.3), now 20 years old, was first diagnosed with Crohn’s when he was 17 years old. He had comparably severe symptoms including lethargy, dizziness, and anorexia. At first, he was diagnosed with tuberculosis and was treated for 9 months. After that, gastrointestinal inflammation recurrence was noticed when a confirmatory endoscopy test was performed. The endoscopic findings were a tight, inflamed terminal ileum and an enterocolonic fistula. Then, after 2 years, a second endoscopic test was done that found severe inflammation at the ascending colon and cecum with anatomical distortion characterized by altered vascularity, congestion (edema), erythema, and pseudopolyps. The findings were worse when compared to previous examinations (Figure 2). He is currently being administered Remicade (Infliximab), which is a chimeric monoclonal antibody used to treat several autoimmune diseases, including IBD.

In family B (Figure 1B), the parents are healthy distant relatives from the same Arabian tribe. In this family, Crohn’s disease was diagnosed in one female and two male siblings. The proband (IV.2) was diagnosed when he was 25 years old and is currently taking Humira (monoclonal antibody). His sister (IV.5) was diagnosed in her late 20s, and she had a colectomy and an ostomy bag. His younger brother (IV.6) was diagnosed when he was 25 years old and was kept on Infliximab (Remicade) for 2 years.

In family C (Figure 1C), the parents are first cousins and healthy, except that the mother has some intestinal inflammation. Interestingly, of the three affected male siblings, two were monozygotic twins. The proband (III.1) was diagnosed 3 years ago, and he is 32 years old now. He has been on Remicade monoclonal antibody treatment every 2 months since the diagnosis. Both twins (III.4 and III.5), now aged 29 years, were diagnosed 6 years ago, and both underwent colectomy at the ages of 26 and 24 years, respectively.

### 3.2. Whole exome sequence analysis

Whole exome sequencing of many family members provided an average of 97,242, 98,011, and 96,297 variants in families A, B, and C, respectively. These massive numbers of variants were further filtered out by excluding 3′ and 5′ UTR variants, conservative and disruptive inframe deletion or insertion, synonymous, intergenic, and intronic variants, coding variants with high allele frequency (>0.01), and poor quality variants with a Phred score of <30. The inclusion of rare coding variants has resulted in 3,498 variants (in 1,455 genes) for family A, 3,721 variants (in 1,571 genes) for family B, and 3,679 variants (in 1,668 genes) for family C. Most of the coding variants in all three families were of the missense type (Table 1). The segregation analysis of the variants in the respective families with IBD did not detect any single rare variant following a classical AR, CH (compound heterozygotes), or *de novo* inheritance pattern. Therefore, we searched for the aggregation of rare variants that would increase the burden status of genes in these families.

**Table 1:** The exome variants yield from siblings of three IBD families.

Case		Total variants	Coding*	Rare**	Number of genes	Homozygous variants	Heterozygous variants
Family A	III.2	98,823	13,300	1721	734	195	1,526
	III.3	95,660	13,157	1777	721	173	1,604
Family B	IV.2	97,925	13,287	1809	785	122	1,687
	IV.5	98,097	13,435	1912	786	150	1762
Family C	III.1	97,737	12,933	1862	833	148	1714
	III.4	94,857	12,796	1817	835	149	1,668

Coding* includes Frameshift, missense, splice acceptor, splice donor, start lost, and stop retained variants.

Rare** (Minor allele frequency < 0.01 in gnomAD, 1,000 genomes, ExAC dbs).

### 3.3. Functional enrichment analysis using immune pathways

The functional enrichment analysis of rare variant genes from individual families revealed a total of 180, 114, and 116 immune-related pathways for families A, B, and C, respectively. In families A, B, and C, 23, 21, and 29 immune pathways respectively were significantly enriched (*p* = <0.05). Table 2 presents the top five significant immune pathways for each family.

**Table 2:** Top five immune system-related pathways enriched in genes with rare coding variants in three families with IBD.

ID	Term	*P*-value*	% Associated genes	Associated genes found
Family A				
GO:0002483	Antigen processing and presentation of endogenous peptide antigen	0.00	38.10	*LEF1, LIG4, PRKDC*
GO:0002697	Regulation of immune effector process	0.01	3.02	*LEF1, LIG4, PRKDC*
GO:0038093	Fc receptor signaling pathway	0.01	2.30	*LILRB1, TMEM176A, TMEM176B*
GO:0045088	Regulation of innate immune response	0.01	8.98	*LIG4, PRKDC, SOS1, SOS2*
GO:0002220	Innate immune response activating cell surface receptor signaling pathway	0.02	11.20	*ERAP1, ERAP2, IDE, SEC14L3*
Family B				
GO:0002250	Adaptive immune response	0.00	2.88	*AHR, BTNL9, CARD9, CD79A, CEACAM1, HLA-B, HLA-C, HLA-DQB1, HLA-DQB2, HLA-DRB1, HLA-DRB5, IL17RA, IRF7, LILRA1, LILRB1, LILRB3, ORAI1, OTUD7B, PDCD1LG2, PPL, RAPGEF3, RASGRP1, RIF1*
GO:0045088	Regulation of innate immune response	0.00	9.58	*A2M, CARD9, CEACAM1, DHX58, HLA-B, IKBKB, IL18RAP, IRF7, KIR2DL4, LILRA2, LILRB1, MUC12, MUC16, MUC17, MUC19, MUC2, MUC20, MUC3A, MUC4, MUC5AC, MUC6, NCR1, NLRC5, OTOP1, PIK3R6, PRKDC, PSMB11, PSME3, PSPC1, RASGRP1, SOCS1, TRIM5*
GO:0045088	Adaptive immune response based on somatic recombination of immune receptors built from immunoglobulin superfamily domains	0.00	2.40	*AHR, CARD9, CEACAM1, HLA-B, HLA-DQB1, HLA-DQB2, HLA-DRB1, IRF7, LILRB1, RIF1*
GO:0042269	Adaptive immune response based on somatic recombination of immune receptors built from immunoglobulin superfamily domains	0.00	18.60	*CEACAM1, HLA-B, IL18RAP, KIR2DL4, LILRB1, NCR1, PIK3R6, RASGRP1*
GO:0002218	Activation of innate immune response	0.00	11.39	*CARD9, IKBKB, LILRA2, MUC12, MUC16, MUC17, MUC19, MUC2, MUC20, MUC3A, MUC4, MUC5AC, MUC6, PRKDC, PSMB11, PSME3, PSPC1, TRIM5*
Family C				
GO:0002220	Innate immune response activating cell surface receptor signaling pathway	0.00	14.40	*CARD9, ICAM3, KLRC2, LILRA2, MUC1, MUC12, MUC16, MUC17, MUC19, MUC2, MUC20, MUC21, MUC3A, MUC4, MUC5AC, MUC5B, MUC6, PSMA8*
GO:0002758	Innate immune response-activating signal transduction	0.00	14.29	*CARD9, ICAM3, KLRC2, LILRA2, MUC1, MUC12, MUC16, MUC17, MUC19, MUC2, MUC20, MUC21, MUC3A, MUC4, MUC5AC, MUC5B, MUC6, PSMA8*
GO:0002223	Stimulatory C-type lectin receptor signaling pathway	0.00	14.05	*CARD9, ICAM3, KLRC2, MUC1, MUC12, MUC16, MUC17, MUC19, MUC2, MUC20, MUC21, MUC3A, MUC4, MUC5AC, MUC5B, MUC6, PSMA8*
GO:0002218	Activation of innate immune response	0.00	12.66	*CARD9, CGAS, ICAM3, KLRC2, LILRA2, MUC1, MUC12, MUC16, MUC17, MUC19, MUC2, MUC20, MUC21, MUC3A, MUC4, MUC5AC, MUC5B, MUC6, PSMA8, PSPC1*
GO:0042113	B cell activation	0.00	2.72	*ATM, HLA-DQB1, HLA-DQB2, IGLC1, IGLL5, LFNG, MSH2, SAMSN1, SLC25A5, YY1AP1*

*P*-value* significant (<0.05).

**Figure 3:**
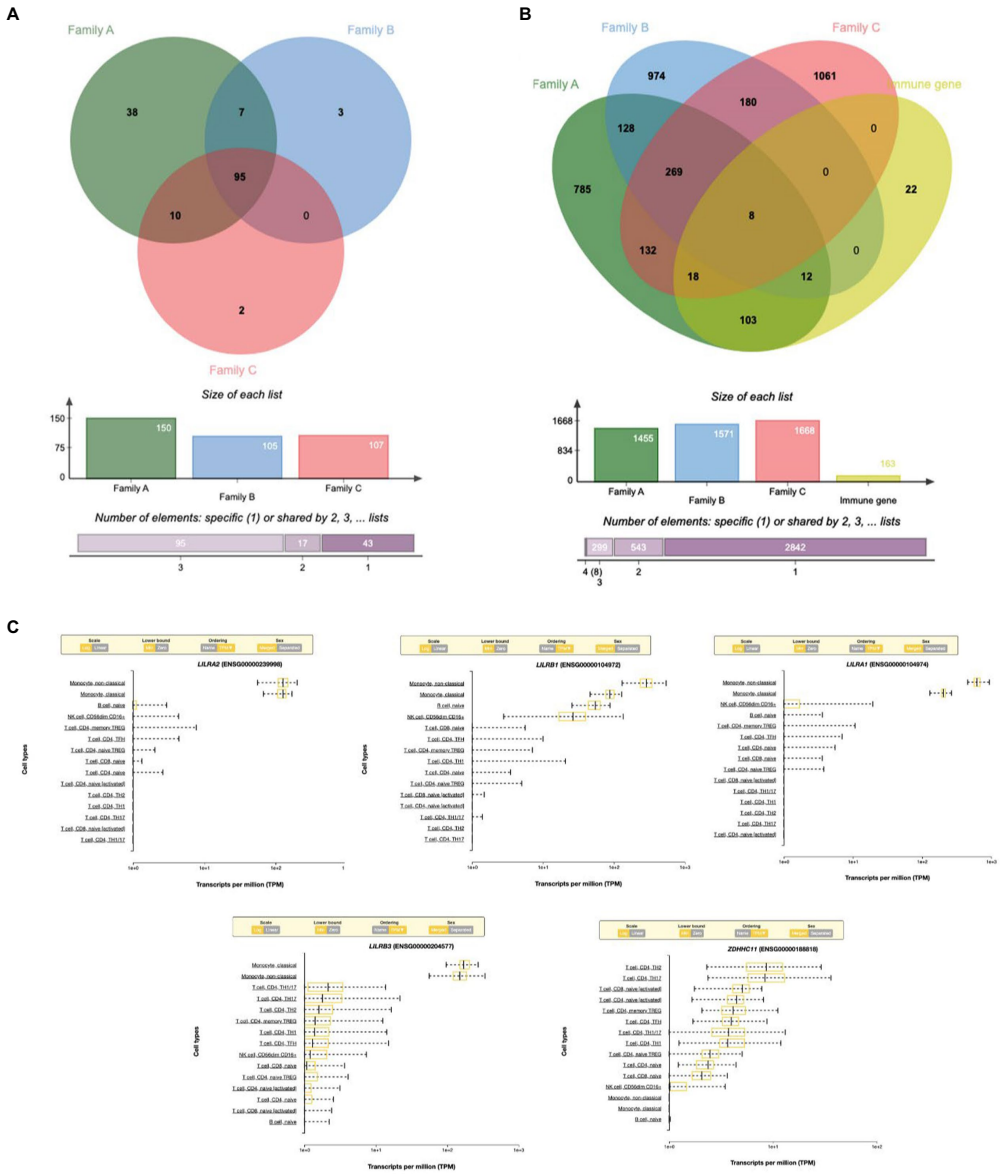
**(A)** Venn diagram showing the shared immune Go-terms between three IBD families. **(B)** Venn diagram showing the shared gene with rare variants between three families. **(C)** Top immune cell gene expression patterns of the genes with rare variants.

A total of 95 (61.3%) GO terms were shared by the three families and analyzed with the VENNY tool. These GO terms were associated with 163 genes after excluding the human leukocyte antigen (*HLA*) complex gene family owing to their known involvement in multiple autoimmune diseases. When we analyzed all 163 genes, only eight genes with rare variants were found to be common among all three families (Figures 3A,B).

### 3.4. Transcript expression analysis of candidate genes in IBD and healthy tissue samples

**Figure 4:**
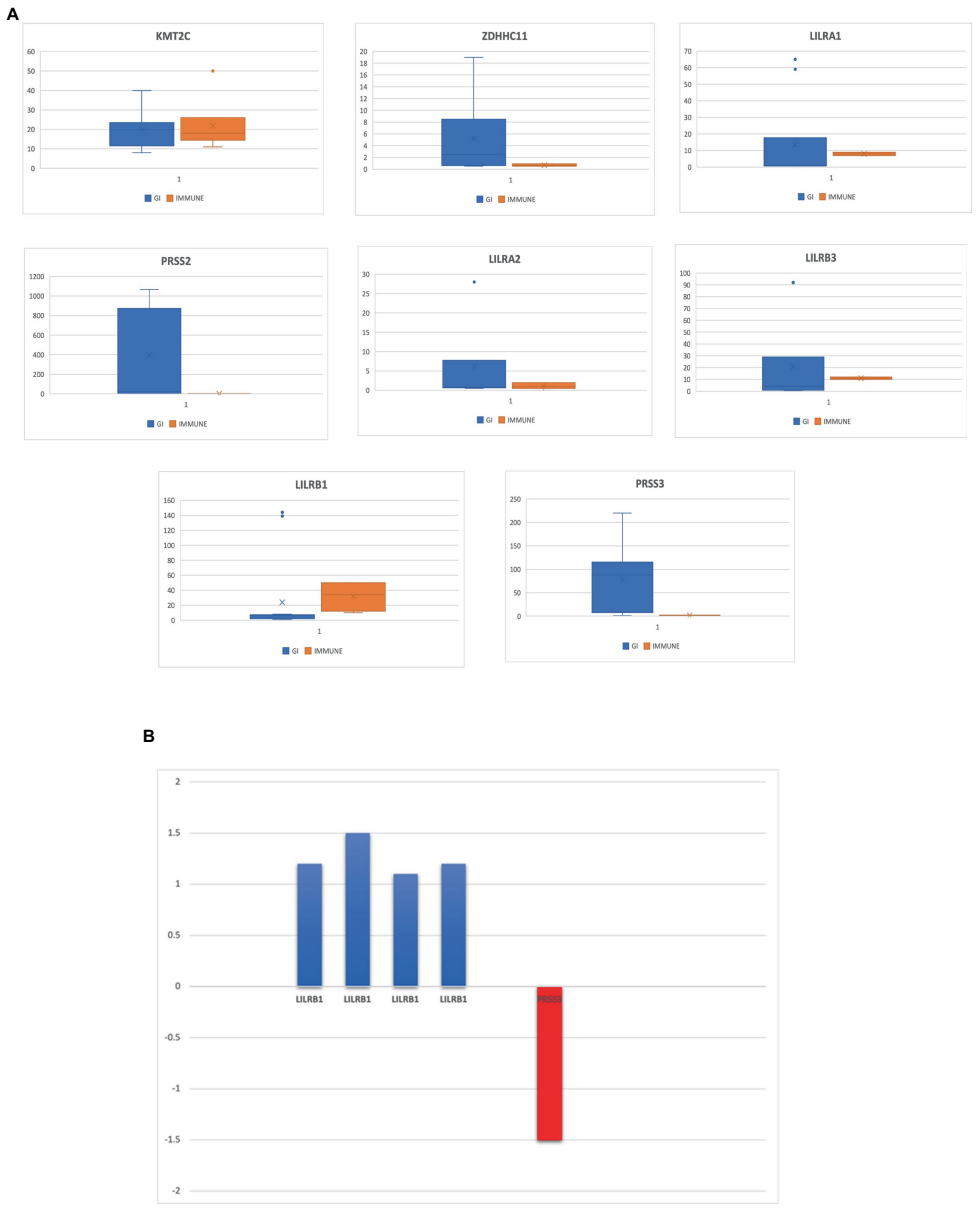
**(A)** Eight genes that are highly expressed in the gastrointestinal tract and several immune tissues. **(B)** Differentially expressed genes in IBD datasets from NCBI-GEO database.

Out of the eight rare variant genes, seven genes were differentially expressed in colonic and mucosal tissues. Of them, two (*ZDHHC11* and *PRSS3*) were downregulated (FC: <−1.1) and five (*LILRB3, LILRA2, LILRB1, PRSS2,* and *LILRA1*) were upregulated. The expression of the upregulated genes (FC: >1.1) is presented in Figure 4B. In the control tissue (gastrointestinal, blood, and immune organs) samples, seven (of the eight) shared genes showed differential expression. *PRSS3* has high expression in the small intestine and colon (Figure 3C).

### 3.5. Immune cell gene expression

Based on RNA sequencing data, we investigated the immune cell type representations of the eight prioritized genes, and only seven genes had significant expression in various immune cells with the log FC of 0.4 (Figure 4A). Leukocyte immunoglobulin-like receptor genes (*LILRB1, LILRB3, LILRA1, and LILRA2*) are highly expressed in immune cells such as monocytes and natural killer (NK) cells. The *LILRB3* gene is highly expressed in classical and non-classical monocytes with an average of 175.84 TPM and 152.3 TPM, respectively. However, this gene is barely expressed in the T cells, with a mean TPM of <2.73. Furthermore*, LILRB1, LILRA1*, and *LILRA2* are highly enriched in non-classical monocytes with means of 290.46, 632.40, and 128.35, respectively, compared to the classical monocytes with an average of 87.91, 204.29, and 123.98, respectively. *KMT2C* is also highly expressed in non-classical monocytes, with a mean of more than 90 TPM (Table 3).

**Table 3:** Summary of the four different computational predictions for potential genes for IBD pathology: normal expression, IBD specific expression, and immune and open target platform.

Gene name	Normal expression (Colon) (average TPM)	IBD specific expression (FC)	Immune (Mean TPM)	Open target platform overall association score
*LILRB1*	2.32	1.25	290.46	0.145
*LILRB3*	1.52	1.825	175.84	<0.1
*KMT2C*	14.6	NA	90.96	0.264
*ZDHHC11*	0.1	−1.1	10.26	<0.1
*LILRA2*	0.3	1.85	128.35	<0.1
*PRSS2*	0	2.23	NA	0.156
*PRSS3*	45.2	−1.5	0.82	0.109
*LILRA1*	0.34	1.575	632.40	<0.1

### 3.6. Open target phenotype identification

From the eight genes identified from the WES rare variant burden analysis, only four genes have shown an association score of >0.1 with gastrointestinal or immune system disease phenotypes. The *KMT2C* gene shared phenotypes with UC with an overall association score of >0.37 (Table 4).

**Table 4:** Number of experimentally validated interactions and predicted interactions for *LILRB1* and *PRSS3* genes from the innate immunity database.

Ensembl gene ID	Organism	Chromosome	Gene symbol	Gene name	Experimentally validated interactions	Interactions predicted by orthology
ENSG00000104972	Homo sapiens	19	*LILRB1*	Leukocyte immunoglobulin-like receptor, subfamily B (with TM and ITIM domains), member 1	12	0
ENSG00000010438	Homo sapiens	9	*PRSS3*	Protease, serine, 3	6	0

### 3.7. Concordance analysis

We used the Venny tool to find genes that were present in both IBD and normal healthy tissue expression analyses, immune cell restricted expression analysis, and open target platform analysis. Of the eight genes, seven (90%) were expressed in IBD tissues, normal healthy tissues (GI, immune organs), and different immune cell types such as monocytes and NK cells. In addition, four genes (50%) showed a strong association (>0.1 score) with gastrointestinal and immune system disease phenotypes. However, all eight genes, *LILRB1, LILRB3, LILRA2, LILRA1, KMT2C, ZDHHC11, PRSS2,* and *PRSS3*, were found to be significant in at least two tools (Table 3).

### 3.8. System biology analysis of innate immunity

Only *LILRB1* and *PRSS3* have physical interactions or associations with the innate immune response in humans, out of the eight genes obtained in the preceding step. *LILRB1* is mapped to chromosome 19, and it has 12 experimentally validated interactions with other genes. Most of the interacting partners are from the *HLA* gene family, such as A, C, G, and F. The gene *PRSS3* interacts with six other genes (Table 4).

### 3.9. Shared genes with rare variants to pathway analysis

These three families shared 10 rare variants for *LILRB1* (ENST00000324602.12) including six missense and four novel frameshift variants. However, 11 unique missense variants were shared only between families B and C. Furthermore, two unique missense variants each were observed in families B and C. Families A and C shared a missense variant in *PRSS3* (ENST00000379405.4) (c.244G > A; rs76740888) and family B had one additional missense variant in *PRSS3* (c.10 T > C; rs772714741) (Table 5).

**Table 5:** Rare variants of *LILRB1* and *PRSS3* genes in three families.

Gene name	Chr. No.	Position	Rs ID	cDNA position	Amino acid position	Effect	MAF	
							1,000 Gp	GenomAD
Family A								
*LILRB1*	19	54,631,583	rs554096090	c.154G > A	p.Gly52Ser	Missense variant	0.001	0.000
*LILRB1*	19	54,631,587	rs199588814	c.158A > T	p.Gln53Leu	Missense variant	0.001	0.000
*LILRB1*	19	54,631,686	rs200880414	c.257C > T	p.Pro86Leu	Missense variant	0.000	0.000
*LILRB1*	19	54,631,724	rs570016342	c.295 T > A	p.Tyr99Asn	Missense variant	0.000	0.000
*LILRB1*	19	54,631,725	rs535742370	c.296A > T	p.Tyr99Phe	Missense variant	0.000	0.000
*LILRB1*	19	54,631,749	rs142396802	c.320G > T	p.Arg107Leu	Missense variant	0.000	0.000
*LILRB1*	19	54,633,154	–	c.1098_1099delAT	p.Trp367fs	Frameshift variant	–	–
*LILRB1*	19	54,633,157	–	c.1100_1101insCT	p.Trp367fs	Frameshift variant	–	–
*LILRB1*	19	54,633,171	–	c.1114_1115insAG	p.Thr372fs	Frameshift variant	–	–
*LILRB1*	19	54,633,173	–	c.1117_1118delTA	p.Tyr373fs	Frameshift variant	–	–
*PRSS3*	9	33,796,675	rs76740888	c.244G > A	p.Val82Ile	Missense variant	–	–
Family B								
*LILRB1*	19	54,631,583	rs554096090	c.154G > A	p.Gly52Ser	Missense variant	0.001	0.000
*LILRB1*	19	54,631,587	rs199588814	c.158A > T	p.Gln53Leu	Missense variant	0.001	0.000
*LILRB1*	19	54,631,605	rs774715846	c.176G > A	p.Arg59His	Missense variant	–	0.000
*LILRB1*	19	54,631,686	rs200880414	c.257C > T	p.Pro86Leu	Missense variant	0.000	0.000
*LILRB1*	19	54,631,724	rs570016342	c.295 T > A	p.Tyr99Asn	Missense variant	0.000	0.000
*LILRB1*	19	54,631,725	rs535742370	c.296A > T	p.Tyr99Phe	Missense variant	0.000	0.000
*LILRB1*	19	54,631,749	rs142396802	c.320G > T	p.Arg107Leu	Missense variant	0.000	0.000
*LILRB1*	19	54,631,944	rs370374304	c.368 T > G	p.Ile123Ser	Missense variant	0.000	0.000
*LILRB1*	19	54,633,033	rs1185911260	c.976G > C	p.Val326Leu	Missense variant	–	–
*LILRB1*	19	54,633,034	rs1486166961	c.977 T > C	p.Val326Ala	Missense variant	–	–
*LILRB1*	19	54,633,037	rs974205214	c.980C > T	p.Ser327Phe	Missense variant	–	0.000
*LILRB1*	19	54,633,049	rs1334566399	c.992A > G	p.Gln331Arg	Missense variant	–	–
*LILRB1*	19	54,633,108	rs765206177	c.1051 T > G	p.Trp351Gly	Missense variant	–	0.000
*LILRB1*	19	54,633,116	rs764221410	c.1059A > C	p.Gln353His	Missense variant	–	0.000
*LILRB1*	19	54,633,150	rs1260040283	c.1093G > T	p.Asp365Tyr	Missense variant	–	–
*LILRB1*	19	54,633,151	rs12985933	c.1094A > C	p.Asp365Ala	Missense variant	–	–
*LILRB1*	19	54,633,154	–	c.1098_1099delAT	p.Trp367fs	Frameshift variant	–	–
*LILRB1*	19	54,633,157	–	c.1100_1101insCT	p.Trp367fs	Frameshift variant	–	–
*LILRB1*	19	54,633,166	rs1401913528	c.1109G > A	p.Arg370Lys	Missense variant	–	–
*LILRB1*	19	54,633,171	–	c.1114_1115insAG	p.Thr372fs	Frameshift variant	–	–
*LILRB1*	19	54,633,173	–	c.1117_1118delTA	p.Tyr373fs	Frameshift variant	–	–
*LILRB1*	19	54,633,185	rs1240220003	c.1128A > T	p.Gln376His	Missense variant	–	–
*LILRB1*	19	54,633,210	rs372567136	c.1153G > A	p.Gly385Ser	Missense variant	–	0.000
*PRSS3*	9	33,795,583	rs772714741	c.10 T > C	p.Phe4Leu	Missense variant	–	–
Family C								
*LILRB1*	19	54,631,583	rs554096090	c.154G > A	p.Gly52Ser	Missense variant	0.001	0.000
*LILRB1*	19	54,631,587	rs199588814	c.158A > T	p.Gln53Leu	Missense variant	0.001	0.000
*LILRB1*	19	54,631,605	rs774715846	c.176G > A	p.Arg59His	Missense variant	–	0.000
*LILRB1*	19	54,631,686	rs200880414	c.257C > T	p.Pro86Leu	Missense variant	0.000	0.000
*LILRB1*	19	54,631,724	rs570016342	c.295 T > A	p.Tyr99Asn	Missense variant	0.000	0.000
*LILRB1*	19	54,631,725	rs535742370	c.296A > T	p.Tyr99Phe	Missense variant	0.000	0.000
*LILRB1*	19	54,631,749	rs142396802	c.320G > T	p.Arg107Leu	Missense variant	0.000	0.000
*LILRB1*	19	54,631,944	rs370374304	c.368 T > G	p.Ile123Ser	Missense variant	0.000	0.000
*LILRB1*	19	54,631,965	rs767704704	c.389A > T	p.Gln130Leu	Missense variant	–	0.000
*LILRB1*	19	54,633,037	rs974205214	c.980C > T	p.Ser327Phe	Missense variant	–	0.000
*LILRB1*	19	54,633,049	rs1334566399	c.992A > G	p.Gln331Arg	Missense variant	–	–
*LILRB1*	19	54,633,108	rs765206177	c.1051 T > G	p.Trp351Gly	Missense variant	–	0.000
*LILRB1*	19	54,633,116	rs764221410	c.1059A > C	p.Gln353His	Missense variant	–	0.000
*LILRB1*	19	54,633,150	rs1260040283	c.1093G > T	p.Asp365Tyr	Missense variant	–	–
*LILRB1*	19	54,633,151	rs12985933	c.1094A > C	p.Asp365Ala	Missense variant	–	–
*LILRB1*	19	54,633,154	–	c.1098_1099delAT	p.Trp367fs	Frameshift variant	–	–
*LILRB1*	19	54,633,157	–	c.1100_1101insCT	p.Trp367fs	Frameshift variant	–	–
*LILRB1*	19	54,633,166	rs1401913528	c.1109G > A	p.Arg370Lys	Missense variant	–	–
*LILRB1*	19	54,633,171	–	c.1114_1115insAG	p.Thr372fs	Frameshift variant	–	–
*LILRB1*	19	54,633,173	–	c.1117_1118delTA	p.Tyr373fs	Frameshift variant	–	–
*LILRB1*	19	54,633,185	rs1240220003	c.1128A > T	p.Gln376His	Missense variant	–	–
*LILRB1*	19	54,633,210	rs372567136	c.1153G > A	p.Gly385Ser	Missense variant	–	0.000
*LILRB1*	19	54,636,536	rs41308744	c.1696G > A	p.Glu566Lys	Missense variant	0.004	0.000
*PRSS3*	9	33,796,675	rs76740888	c.244G > A	p.Val82Ile	Missense variant	–	–

These two genes, *LILRB1* and *PRSS3*, were studied independently to map the biochemical pathways associated with them. Our findings showed that *LILRB1* is connected to three pathways, namely, the adaptive immune system, the immune system, and immunoregulatory interactions between a lymphoid and a non-lymphoid cell. The *PRSS3* gene is involved in 10 different pathways, namely, neutrophil degranulation, the innate immune system, antimicrobial peptides, the metabolism of vitamins and cofactors, the metabolism of water-soluble vitamins and cofactors, alpha-defensins, defensins, the immune system, and cobalamin (Cbl, vitamin B12) transport and metabolism.

### 3.10. Gene–gene networking analysis

Many of the physically interacting partners of *PRSS3* (such as *TCN1, DEFA4, DEFA1, DEFA5, DEFA3, DEFA6, PRSS2, SPINK1,* and *CBLIF*) are co-regulated and co-expressed with interacting partners of *LILRB1* (*HLA-B, LILRA1,* and *LILRA3*). Indirect dysregulated interactions between many of these proteins might trigger inflammation in IBD (Table 6).

**Table 6:** Protein–protein interactions of *LILRB1* and *PRSS3* genes.

Interactors	Species	Type	Source database ID(s)	Interactor types	Tissue
LILRB1 with HLA-B	Homo sapiens	Physical interaction	BIOGRID-256234	Protein – protein	–
LILRB1 with HLA-A	Homo sapiens	Association	IDB-120686	Protein – protein	Kidney cell line
CSK with LILRB1	Homo sapiens	Association	MINT-8027327; EBI-7351403	Protein – protein	–
HLA-F with LILRB1	Homo sapiens	Physical interaction	BIOGRID-276645	Protein – protein	–
LILRB1 with HLA-A	Homo sapiens	Association	BIOGRID-255783	Protein – protein	–
PTPN6 with LILRB1	Homo sapiens	Physical association	IDB-190120; BIOGRID-318101	Protein – protein	–
CSK with LILRB1	Homo sapiens	Physical interaction	IDB-117837; IDB-117834	Protein – protein	T-lymphocyte cell line
LILRB1 with HLA-C	Homo sapiens	Physical interaction	BIOGRID-256235	Protein – protein	–
LILRB1 with HLA-G	Homo sapiens	Physical interaction	BIOGRID-256236; MINT-7144982; EBI-7087620	Protein – protein	–
PTPN6 with LILRB1	Homo sapiens	Physical interaction	IDB-117838; IDB-117836	Protein – protein	T-lymphocyte cell line
B2M with LILRB1	Homo sapiens	Association	BIND-121495	Protein – protein	–
CSK with LILRB1	Homo sapiens	Association	EBI-7351451; MINT-8027342	Protein – protein	–
PRSS3 with SERPINA1	Homo sapiens	Association	BIND-117882; BIND-90568	Protein – protein	–
Complex of 10 interactors	Homo sapiens	Association	EBI-8770525	Protein – protein	–
PRSS3 with ALB	Homo sapiens	Association	BIOGRID-825632	Protein – protein	–
PRSS3 with HDGF	Homo sapiens	Association	BIOGRID-635705	Protein – protein	–
TFPI with PRSS3	Homo sapiens	Association	BIOGRID-317015	Protein – protein	–
PRSS3 with UBC	Homo sapiens	Association	BIOGRID-627754; BIOGRID-618329	Protein – protein	–

### 3.11. Amino acid conserved domains

A crucial step in determining the relationship between the nucleotide sequence, protein structure, and function of disease-causing proteins is by mapping the conserved amino acid domains. According to the CDD analysis, the *LILRB1* protein contains an immunoglobulin (Ig) superfamily domain located between 28 and 419 amino acid positions (four domains). *PRSS3* protein consists of a Trypsin-like serine protease domain between 38 and 256 amino acids. We excluded variants that were located outside the conserved domains area (Table 7).

**Table 7:** Conserved domains and their amino acid locations in *LILRB1* and *PRSS3*.

Gene	cDNA position	Amino acid location	Domain	Domain range
*LILRB1*	c.154G > A	p.Gly52Ser	IgC2_D1_LILR_KIR_like	28–118
*LILRB1*	c.158A > T	p.Gln53Leu	IgC2_D1_LILR_KIR_like	28–118
*LILRB1*	c.176G > A	p.Arg59His	IgC2_D1_LILR_KIR_like	28–118
*LILRB1*	c.257C > T	p.Pro86Leu	IgC2_D1_LILR_KIR_like	28–118
*LILRB1*	c.295 T > A	p.Tyr99Asn	IgC2_D1_LILR_KIR_like	28–118
*LILRB1*	c.296A > T	p.Tyr99Phe	IgC2_D1_LILR_KIR_like	28–118
*LILRB1*	c.320G > T	p.Arg107Leu	IgC2_D1_LILR_KIR_like	28–118
*LILRB1*	c.389A > T	p.Gln130Leu	IgC2_D1_LILR_KIR_like	28–118
*LILRB1*	c.1098_1099delAT	p.Trp367fs	Ig super family	327–419
*LILRB1*	c.1100_1101insCT	p.Trp367fs	Ig super family	327–419
*LILRB1*	c.1114_1115insAG	p.Thr372fs	Ig super family	327–419
*LILRB1*	c.980C > T	p.Ser327Phe	Ig super family	327–419
*LILRB1*	c.992A > G	p.Gln331Arg	Ig super family	327–419
*LILRB1*	c.1051 T > G	p.Trp351Gly	Ig super family	327–419
*LILRB1*	c.1059A > C	p.Gln353His	Ig super family	327–419
*LILRB1*	c.1093G > T	p.Asp365Tyr	Ig super family	327–419
*LILRB1*	c.1094A > C	p.Asp365Ala	Ig super family	327–419
*LILRB1*	c.1117_1118delTA	p.Tyr373fs	Ig super family	327–419
*LILRB1*	c.1128A > T	p.Gln376His	Ig super family	327–419
*LILRB1*	c.1153G > A	p.Gly385Ser	Ig super family	327–419
*PRSS3*	c.10 T > C	p.Phe4Leu	Trypsin-like serine protease	38–256
*PRSS3*	c.244G > A	p.Val82Ile	Trypsin-like serine protease	38–256

### 3.12. *LILRB1* and *PRSS3* 3D model construction

The predicted 3D protein structure was collected from Alphafold, the state-of-the-art AI system developed by DeepMind, and I-TASSER. The total length (650 aa) of the structures of human *LILRB1* protein chain A, with model confidence (pLDDT >70), was downloaded as a PDB file. The full-length (247 aa) structure model of human *PRSS3* protein chain A was downloaded as a PDB file, with a model confidence score (C-score of −0.54), an estimated TM score of 0.64 ± 0.13 Å, and an estimated RMSD = 6.9 ± 4.1 Å. The *LILRB1* (p.Gln53Leu, p.Tyr99Asn, p.Trp351Gly, p.Asp365Ala, and p.Gln376His) and *PRSS3* (p.Phe4Leu and p.Val25Ile) were then modeled using homology modeling by the SWISS-MODEL using energy-minimized native protein structures.

### 3.13. Protein stability analysis

**Figure 5:**
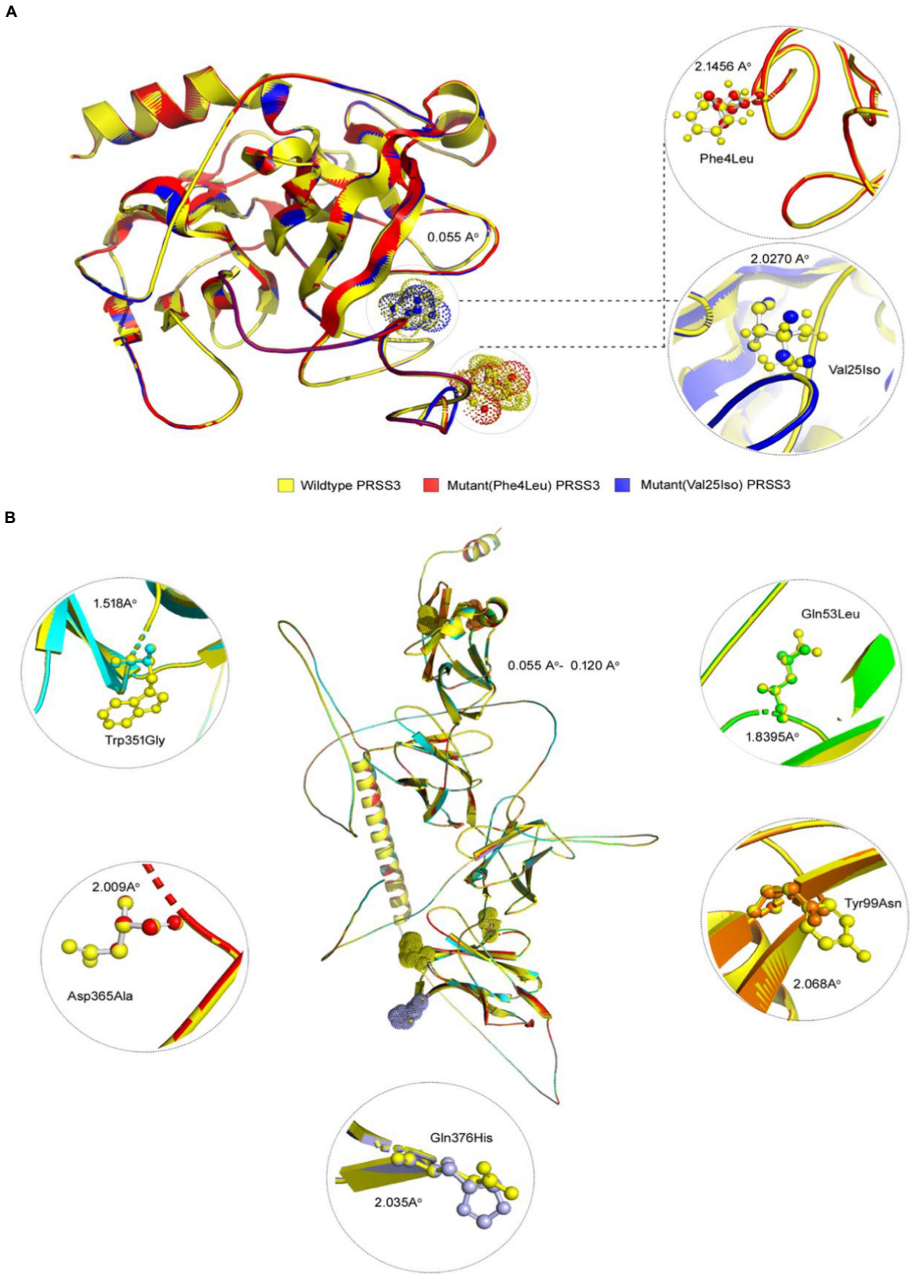
3D structures of *PRSS3* and *LILRB1* wild and mutant protein models. Structures of **(A)** *PRSS3* wild type in yellow, and mutant (p.Phe4Leu and p.Val25Ile) in red and blue, respectively **(B)** *LILRB1* wild type in yellow, and mutant (p.Gln53Leu) in green (p.Tyr99Asn) orange (p.Trp351Gly) blue (p.Asp365Ala), red, and (p.Gln376His) purple.

Pathogenic amino acid substitutions can result in changes in free energy values, thereby directly impacting protein stability. We analyzed the impact of 16 *LILRB1* (G52S, Q53L, R59H, P86L, Y99N, Y99F, R107L, Q130L, S327F, Q331R, W351G, Q353H, D365Y, D365A, Q376H, and 2 G385S) and *PRSS3* (F4L and V25I) variants on protein stability by MAESTRO. MAESTRO is a robust tool for predicting stability changes following point mutations by providing predicted free energy change (ΔΔG) values and a corresponding prediction confidence estimation (c_pred_). For the *LILRB1* protein, out of the 16 variants, only five had a destabilizing effect on the protein (Q53L, Y99N, W351G, D365Y, and D365A) with ΔΔG of 0.074, 0.85, 0.380, 0.083, and 0.086, respectively. The c_pred_ scores were 0.088, 0.923, 0.875, 0.885, and 0.872, respectively. The two *PRSS3* (F4L and V25I) variants had a destabilizing effect with ΔΔG of 0.016 and 0.799 and c_pred_ of 0.885 and 0.857 (Table 8). We used the YASARA tool to analyze the native and mutant *LILRB1* and *PRSS3* structures to evaluate their structural drifts (in terms of RMSD at residue and whole protein levels). The RMSD value is utilized to quantify the structural similarity between two atomic coordinates when they are superimposed. When there is divergence at the polypeptide chain level, impact of substitution mutations on amino acid structures can be determined. For the *LILRB1* protein, the five substitutions with destabilizing effects on the protein (Q53L, Y99N, W351G, D365Y, and D365A) had RMSDs at residue levels of 1.8395, 2.0688, 1.5186, 2.0098, and 2.0351. The two *PRSS3* (F4L and V25I) variants with destabilizing effects had RMSD at residue levels of 2.1465 and 2.0270, respectively (Figure 5 and Table 9).

**Table 8:** MAESTRO program protein stability prediction on *LILRB1* and *PRSS3* variants.

Substitutions	Gene name	ΔΔG_pred_ (kcal/mol)	c_pred (kcal/mol)_
G52.A(S)	*LILRB1*	−0.170	0.909
Q53.A(L)	*LILRB1*	0.074	0.880
R59.A(H)	*LILRB1*	−0.154	0.919
P86.A(L)	*LILRB1*	−0.363	0.885
Y99.A(N)	*LILRB1*	0.85	0.923
Y99.A(F)	*LILRB1*	−0.112	0.903
R107.A(L)	*LILRB1*	−0.607	0.864
Q130.A(L)	*LILRB1*	−0.132	0.916
S327.A(F)	*LILRB1*	−0.438	0.860
Q331.A(R)	*LILRB1*	−0.063	0.873
W351.A(G)	*LILRB1*	0.380	0.875
Q353.A(H)	*LILRB1*	−0.047	0.911
D365.A(Y)	*LILRB1*	−0.224	0.881
D365.A(A)	*LILRB1*	0.083	0.885
Q376.A(H)	*LILRB1*	0.086	0.872
G385.A(S)	*LILRB1*	−0.169	0.865
F4.A(L)	*PRSS3*	0.016	0.885
V25.A(I)	*PRSS3*	0.799	0.857

ΔΔG Positive score, Destabilizing; Negative score, Stabilizing.

**Table 9:** YASARA program RMSD at residue and whole level.

Gene name	Substitutions	Calpha RMSD (Å)	RMSD (Å)
*LILRB1*	Q53.A(L)	0.055	1.8395
*LILRB1*	Y99.A(N)	0.055	2.0688
*LILRB1*	W351.A(G)	0.055	1.5186
*LILRB1*	D365.A(A)	0.055	2.0098
*LILRB1*	Q376.A(H)	0.055	2.0351
*PRSS3*	F4.A(L)	0.121	2.1456
*PRSS3*	V25.A(I)	0.120	2.0270

Cutoff RMSD at the polypeptide chain > 0.2, residue levels > 2.

## 4. Discussion

Most genetic studies on IBD have largely concentrated on identifying common variants with small effect sizes through GWAS studies (9). However, rare and highly penetrant variations identified through population-specific cohorts or family-focused research have immense potential to catch the variants with high effect size on complex diseases such as IBD (8, 12). Although, studying the familial cases may uncover rare causal variants, their heritability of disease in unrelated patient cohorts is still uncertain (23). Unlike VEO-IBD, which has a causal monogenic factor, late-onset is a complex and multifactorial disorder that cannot be explained by classical genetic segregation methods (16, 24, 25). Large-scale sporadic case–control studies on WES-based rare variant burden analysis (RVB) have previously identified several strong risk loci for complex diseases, such as Schizophrenia (26), Alzheimer’s disease (27), epilepsy (2019), autism (28), and Crohn’s disease (12).

According to a recent systematic review and meta-analysis of IBD in the Arab World, the consanguinity rate in Saudi Arabian IBD patients is as high as 32.6% (4). Consanguinity acts as a prerequisite risk factor for several autosomal recessive immune disorders (29, 30). Therefore, identifying the actual genetic causes underlying familial IBD is expected to aid in early detection, therapy optimization, carrier screening, and genetic counseling for extended families. In this context, we have sequenced the exomes of three consanguineous Saudi families with more than one IBD-affected sibling. We performed segregation analyses of the variants in the respective IBD families. However, this did not result in identifying any causal rare variant fitting into the classical autosomal recessive, compound heterozygote, or *de novo* inheritance patterns. Since the classical Mendelian segregation analysis does not apply to all forms of IBD, single-gene models often fail to explain the complex molecular etiology of the disease. For example, in other gastrointestinal diseases, such as celiac disease (CeD), a recent study of two rare Arab families with CeD concluded that the genetic variability cannot be explained by classical genetic segregation techniques, because the single gene model is incapable of dissecting the disease’s molecular elements (24). It has adopted multidimensional computational analysis to identify and characterize the potential autoimmunity risk genes for Celiac disease (19). Therefore, following a similar strategy, we searched and identified potential IBD genes based on the rare variant burden analysis using a combination of artificial intelligence approaches, bioinformatic tools, and multi-dimensional, large-scale next-generation sequence datasets. This novel approach at a large scale is likely to provide some valuable clues to novel biomarkers or drug targets for many complex diseases in the future (24, 31–34).

We prioritized from thousands of rare variants of WES to potential two candidate genes, *LILRB1* and *PRSS3*, owing to their strong involvement in the innate immune system. Both genes are linked to inflammation, a process in which multiple pathways interact to contribute to this complex function. The *LILRB1* gene is a member of the leukocyte immunoglobulin-like receptor (*LILRs*; or *ILT, LIR,* and *CD85*) family, which are the most conserved genes located within the leukocyte receptor cluster on human chromosome 19 (35, 36). The family consists of 13 members with activating or inhibitory properties: *LILRs* with long cytoplasmic tails that contain inhibitory motifs based on tyrosine act as inhibitory receptors (*LILRBs*), whereas *LILRs* with short cytoplasmic tails act as activators (*LILRAs*). *LILRs* are two pseudogenes and 11 functional genes encoding five activating (*LILRA1, 2, 4–6*), five inhibitory (*LILRB1–5*), and one soluble form (*LILRA3*). Moreover, *LILRs* are classified into two classes based on the amino acid sequence similarity of the region that binds to *HLA. LILRB1, B2, A1, A2,* and *A3* are classified as members of group 1 that are highly similar in sequence and are likely to interact with *HLA* class I molecules (*HLAIs*). Furthermore, *LILRB1* has been shown to inhibit the combination of *CD8* and *HLA I* molecules, hence regulating *CD8*+ T cells (37, 38).

From our results, we found that the three families shared 10 rare variants (six missense and four novel frameshift variants) in the *LILRB1* gene. However, 11 unique missense variants were shared only between families B and C. Furthermore, two unique missense variants were shown in families B and C, respectively. Of these variants, five (p.Gln53Leu; p.Tyr99Asn; p.Trp351Gly; p.Asp365Ala; and p.Gln376His) were seen to have a destabilizing effect on the corresponding protein with ΔΔG upon mutations of 0.074, 0.85, 0.380, 0,083, and 0.086 (kcal/mol), respectively, and the c_pred_ upon mutations of 0.088, 0.923, 0.875, 0.885, and 0.872 (kcal/mol) respectively. All these variants were rare and not present in public databases such as the Greater Middle East (GME), the KAIMRC Genomic Database (KGD), and the Genome Aggregation Database (gnomAD) (16, 39). Various *LILRB1* rare variants seen in these families might be dysregulating several immune pathways, such as adaptive immunity, that normally prevent pathogens from growing by specialized, systemic cells and processes (40). Another important pathway is the immunoregulatory interactions between a lymphoid and a non-lymphoid cell. A variety of receptors and cell adhesion molecules play important roles in modifying the response of lymphoid cells (such as B-, T-, and NK cells) to self, tumor antigens, and pathogenic organisms (41). Since the innate immune system detects microbial infections, any defect in this system could lead to microbial imbalance that could trigger IBD development.

The second gene, *PRSS3,* is a member of the trypsin family of serine proteases (synonyms: *PRSS4, TRY3*, and *TRY4*). This enzyme is found in the brain and pancreas, and it is resistant to common trypsin inhibitors. It acts on peptide bonds containing the carboxyl group of lysine or arginine. This gene is located on chromosome 9 at the locus of the T cell receptor beta variable orphans. *The PRSS3* gene has four transcripts encoding distinct isoforms. Furthermore, this gene is a known contributor to the initiation and progression of malignant tumors, but its significance in gastric cancer (GC) remains unknown (42). This is the first report linking the novel potential role of the *PRSS3* gene to IBD through shared rare variant burden analysis in three families from Saudi Arabia presenting late-onset IBD.

In the present study, we found that both families A and C shared the same missense variant for *PRSS3* (c.244G > A; rs76740888). Family B had a missense variant for *PRSS3* (c.10 T > C; rs772714741). The frequency of the *PRSS3*, c.244G > A variant in the GME variome project is 11%, which might be seen only among the Arab population. However, this variant is not present in gnomAD. Moreover, two prediction tools, the Mutation Taster and the likelihood ratio test (LRT), show that this variant is damaging. The frequency of the c.10 T > C variant is rare and not present among GME, KGD, and gnomAD. Interestingly, both variants have a destabilizing effect on the protein structure, with ΔΔG of 0.016 and 0.799 (kcal/mol) and c_pred_ of 0.885 and 0.857 (kcal/mol) (43). Destabilizing mutations reduce the stability of a protein and may lead to its misfolding, aggregation, and degradation (44).

Different rare variants of the *PRSS3* gene might be perturbing several immune pathways, such as the innate immune system and neutrophil degranulation (45). Any defect in these important pathways could harm autoimmunity, which will lead to the development of any disease linked to autoimmunity, such as IBD. Our findings suggest a novel strategy for deciphering the complex genetic basis of IBD through the whole exome sequence (WES) analysis of familial cases combined with computational analysis. This study was performed on three consanguineous Saudi families with IBD with each family having more than one affected sibling.

We sincerely acknowledge some limitations of this study. First, our findings were limited to three families with IBD, and studying more familial cases will help establish the role of the *LILRB1* and *PRSS3* and other potential causal genes, biomarkers, and drug targets for IBD. But our findings could be a proof of concept that rare variant burden (RVB) can assist in unraveling the genetic complexity of IBD, where classical Mendelian segregation models are of limited use. Second, while our study was conducted on humans, studying the role of LILRB1 and PRSS3 genetic variants on intestinal cell lines and animal models could aid in understanding how mutant proteins modulate autoimmune responses at the tissue level. Third, computational methods often show variable predictions; hence, their results should be interpreted in the context of subsequent biological experiment-based verifications.

## 5. Conclusion

This study proposes a novel strategy for understanding the genetic complexity of IBD by combining WES and computational multi-dimensional biological data analysis to identify potential IBD key proteins. Our findings suggest that the rare and novel variants identified in two potential key proteins (*LILRB1* and *PRSS3*) are likely to contribute to IBD pathogenesis *via* several important immune pathways, such as the innate and adaptive immune system pathways and neutrophil degranulation.

## Data availability statement

The datasets presented in this article are not readily available because (a) participants’ refusal to store or distribute the genomic data in the public domain and (b) as per the local Institutional Ethics Committee approval and national policy on genomic data sharing in the public domain outside the country. Allowed data under the above mentioned restrictions of the IRB and participants requirements is presented in the article as well in the supplementary material, further inquiries can be directed to the corresponding authors.

## Ethics statement

The studies involving human participants were reviewed and approved by the Institutional Review Board (IRB) protocols at King Abdulaziz University Hospital. The patients/participants provided their written informed consent to participate in this study.

## Author contributions

RJ, RE, and NS: conceptualization and writing—original draft preparation. RJ, ZA, BB, and RE: methodology. RJ and BB: software and visualization. RJ, BB, RE, and NS: formal analysis. BB and NS: resources. RJ, HA-N, ZA, NA-T, NA, HA, MA, BA, NS, YQ, OS, BB, MM, and RE: writing—review and editing. NS, OS, BB, and RE: supervision. RE: project administration and funding acquisition. All authors contributed to the article and approved the submitted version.

## Funding

The authors extend their appreciation to the Deputyship for Research & Innovation, Ministry of Education in Saudi Arabia for funding this research work through the project number (IFPHI-130-140-2020) and King Abdulaziz University, DSR, Jeddah, Saudi Arabia.

## Conflict of interest

The authors declare that the research was conducted in the absence of any commercial or financial relationships that could be construed as a potential conflict of interest.

## Publisher’s note

All claims expressed in this article are solely those of the authors and do not necessarily represent those of their affiliated organizations, or those of the publisher, the editors and the reviewers. Any product that may be evaluated in this article, or claim that may be made by its manufacturer, is not guaranteed or endorsed by the publisher.

## Footnotes

^1^https://bioinfogp.cnb.csic.es/tools/venny/

^2^https://www.ebi.ac.uk/gxa/home

^3^https://dice-database.org/

^4^https://platform.opentargets.org/

^5^https://www.innatedb.com/

^6^https://alphafold.ebi.ac.uk/

^7^https://pymol.org/2/
